# A Quaternary Code Correcting a Burst of at Most Two Deletion or Insertion Errors in DNA Storage

**DOI:** 10.3390/e23121592

**Published:** 2021-11-27

**Authors:** Thi-Huong Khuat, Sunghwan Kim

**Affiliations:** Department of Electrical, Electronic and Computer Engineering, University of Ulsan, Ulsan 44610, Korea; khuathuong090887@gmail.com

**Keywords:** DNA storage, quaternary code, deletion error, insertion error, consecutive errors

## Abstract

Due to the properties of DNA data storage, the errors that occur in DNA strands make error correction an important and challenging task. In this paper, a new code design of quaternary code suitable for DNA storage is proposed to correct at most two consecutive deletion or insertion errors. The decoding algorithms of the proposed codes are also presented when one and two deletion or insertion errors occur, and it is proved that the proposed code can correct at most two consecutive errors. Moreover, the lower and upper bounds on the cardinality of the proposed quaternary codes are also evaluated, then the redundancy of the proposed code is provided as roughly $2\log_{4}8n$.

## 1. Introduction

In recent years, because of its huge capacity and excellent durability, deoxyribonucleic acid (DNA) storage is becoming attractive for future long-term data storage [1,2,3]. However, during the processes of DNA storage, the molecule can be faced with errors that do not normally occur in traditional storage devices such as deletion and insertion errors [4]. Therefore, research to address deletion and insertion errors is extremely significant in DNA storage, and error-correcting codes for the errors have been studied. Our work focuses on the codes capable of correcting multiple deletion or insertion errors in DNA storage.

For correcting one deletion or insertion error in binary codes, Varshamov–Tenengolts (VT) codes were first proposed in [5] and in the same year the modified VT code construction was provided in [6] to correct a single deletion, insertion or substitution error. Shortly thereafter, to deal with more than a single error, Levenshtein extended the VT code to a binary code that can correct at most two consecutive deletion or insertion errors [7]. In [8], a binary codeword was arranged as an array with *b* rows and each row was a binary VT codeword so that this construction could correct a burst of the size of exactly *b* deletion or insertion errors (with any fixed b≥2). Then, the authors of [9] proposed a binary shifted-Varshamov–Tenengolts (SVT) code to obtain an improved construction which still corrects exactly *b* errors but with a lower redundancy than one in [8]. From the obviously efficient correction and low redundancy of the VT codes, the authors in [10,11] proposed a method of the linear-time encoders to implement the binary VT code which satisfies the homopolymer run and Guanine-Cytosine(GC)-content constraints [12,13] among important properties of a DNA strand. However, the binary VT codes used in these linear-time encoders correct a single nucleotide of a DNA strand. With a similar approach as [10,11], but to correct a burst of size exactly *b* deletions or insertions of DNA symbols, the authors of [14] applied the encoder of the binary modified VT code in [6] and binary SVT codes in [9]. Then, by interleaving bits of binary VT codewords and binary SVT codewords, the work [9] obtained a binary code construction that can correct a burst error of size exactly 2b, and finally, the codeword of this construction was translated to DNA symbols.

A non-binary VT code was first proposed in [15], and a non-binary SVT code was proposed in [16]. The codes were defined over a *q*-ary alphabet for any q>2. With the similar property of the binary codes, the *q*-ary VT and *q*-ary SVT codes can correct a single deletion or insertion symbol. To correct multiple errors, the construction in [8,9] can be applied to obtain a *q*-ary code that can correct a burst of size exactly *b* of deletion or insertion errors. However, designing *q*-ary VT codes that can correct multiple deletion or insertion errors has been an interesting problem [17]. Recently, there were some works [18,19,20,21] focused on code design to correct exact multiple errors but the efficient design for *q*-ary codes (or even quaternary codes) that can correct a burst of at most *b* deletion or insertion errors is still an open problem. The authors of [22] proposed a non-binary code correcting at most two consecutive deletions with redundancy $\log n+\log q\left(\log\left(\log n+6\right)+\log6\right)+3$. In [22], the authors used the construction method in [9] with one binary code in [7] and a modified of it in interval *P*. However, we propose a quaternary code which is suitable for robust DNA storage and can correct at most two consecutive deletion or insertion errors with the direct construction. Moreover, the redundancy of the proposed code is improved than [22].

As to the cardinality of VT codes, for about 50 years, a lower bound of size of the best class of VT codes can be achieved, but an upper bound is rarely provided even in binary case. The author in [23] used Mixed Integer Linear Programming (MILP) relaxation technique to obtain the tighter upper bound of the binary VT code, for example, with the length *n* = 11, the maximum size of one deletion code was calculated as 173. Moreover, the conjecture about maximum size of VT code for all *n* was also provided. However, in this work, we focus on the correction error capability of the proposed code design, then we use the previous methods in [7,15] to evaluate lower bound and upper bound of the proposed code design.

In our work, we have extended binary codes based on the results of [7], by adding two constraints to determine the exact values and positions of the errors in the quaternary sequence. By mathematically analyzing the possible cases of errors, we propose decoding algorithms to prove the error correction capability of this code design. We note that the main concern in this work is the error correction capability of quaternary code design, not focus on constraints in DNA storage. It is assumed that the combination design of error correction code and constraints of DNA storage was already done by other algorithms [11,24]. The main contributions in this paper can be summarized as follows.

- We propose a quaternary code design that is suitable for the deletion or insertion channel, especially for mapping 0↔A, 1↔C, 2↔T, and 3↔G. This proposed design is directly applicable to sequencing in DNA storage. Furthermore, this proposed code can correct at most two consecutive deletion or insertion errors.
- We propose two decoding algorithms for this proposed code to correct one deletion and two consecutive deletion errors. For the decoding of insertion errors, some differences between the deletion and insertion cases are shown and the important functions for correcting the insertion error are also presented in Appendix A.
- We provide the lower bound and evaluate upper bound of the proposed code design. The redundancy of the proposed code design is also calculated to be at most $2\log_{4}8n$.

This paper is organized as follows. In Section 2, we list basic notations and definitions used in the rest of the paper and we briefly present previous binary and quaternary code constructions to correct one and two consecutive deletions. Then, Section 3 contains the proposed code construction, a proof of the correction capability, and the bounds of the cardinality for the proposed quaternary code. Section 4 provides a discussion and, finally, conclusion is presented in Section 5 of this paper.

## 2. Preliminaries and Previous Works

### 2.1. Notation and Definition

Let $F_{2}^{n}$ and $F_{4}^{n}$ be the set of binary and quaternary sequences of length *n*, respectively. Let a quaternary codeword with length *n* be defined as c = $\left(c_{1},c_{2},\ldots ,c_{n}\right)\in F_{4}^{n}$. Then, a modified sequence $c_{l}^{n-b}$ of the sequence c is defined as $c_{l}^{n-b}$ = $\left(c_{1},c_{2},\ldots ,c_{l-2},c_{l-1},c_{l+b},c_{l+b+1},\ldots ,c_{n}\right)\in F_{4}^{n-b}$, where $c_{l},c_{l+1},\ldots ,c_{l+b-1}$ are deleted in c. Similarly, a sequence $c_{l}^{n+b}$ of the sequence c is also defined as $c_{l}^{n+b}$ = $\left(c_{1},c_{2},\ldots ,c_{l-1},h_{1},h_{2},\ldots ,h_{b},c_{l},c_{l+1},\ldots ,c_{n}\right)\in F_{4}^{n+b}$, where $h_{1},h_{2},\ldots ,h_{b}$ are inserted from *l*-th position in c.

For a binary sequence x = $\left(x_{1},x_{2},\ldots ,x_{n}\right)\in F_{n}^{2}$, we can consider a sequence 0x with length n+1, where 0x = $\left(0,x_{1},x_{2},\ldots ,x_{n}\right)\in F_{2}^{n+1}$. For simplicity, the sequence 0x with length n+1 is regarded as having a starting value of $x_{0}$ = 0. For example, a binary sequence x with length 10 is given as x = (0,0,1,0,0,1,1,1,1,1). For convenience, the binary sequence notation can be changed to x = 0010011111. In the rest of this paper, these two notations are used as the same meaning, so there is a binary sequence 0x, with length 11, as 0x = 00010011111. Then, the run-length vector r denotes the number of zeros and ones run-length in 0x. In addition, the binary sequence 0x is composed of four runs $u_{0}u_{1}u_{2}u_{3}$, which are $u_{0}$= 000, $u_{1}=1$, $u_{2}$= 00, and $u_{3}$= 11111. Herein, for a non-negative integer *k*, the zeros and ones runs are denoted $u_{2k}$ and $u_{2k+1}$, respectively. Then, the run-length vector r of 0x is r = $\left(r_{0},r_{1},r_{2},r_{3}\right)$= (3,1,2,5).

Let ∥r∥ be the total number of elements in the run-length vector r of 0x, corresponding to the total number of runs in 0x. Then, from the run-length vector, the run-syndrome of the binary sequence 0x is defined as

(1) $$\begin{array}{c} Rsyn\left(0x\right)=\sum_{i=0}^{∥r∥-1}ir_{i}. \end{array}$$

In the previous example, for 0x = 00010011111, since the run-length vector r is (3,1,2,5), Rsyn(0x) = $\sum_{i=0}^{4-1}ir_{i}$= 20.

If the *j*-th bit of 0x belongs to the *m*-th run $u_{m}$, we define $k_{0x}\left(j\right)$ as the index of the run and $k_{0x}\left(j\right)=m$, for 1≤j≤n. Since the total number of elements of the run-length vector cannot exceed the length of 0x, ∥r∥ is bounded as

(2) $$\begin{array}{c} ∥r∥\leq n+1, \end{array}$$

where the equality is satisfied if the binary sequence 0x = 0101010⋯.

From the previous example, the binary sequence 0 = 00010011111, with length 11, has the run-length vector r = $\left(r_{0},r_{1},r_{2},r_{3}\right)$ = (3,1,2,5) and the total number of elements of the run-length vector of 0x is ∥r∥ = 4 <n+1= 11. For 1≤j≤10, since the third bit in 0x belongs to the run $u_{1}$ =1, the index of the run which the third bit belongs to is $k_{0x}\left(3\right)$ = 1.

### 2.2. Previous Works

With the binary case, to the best of our knowledge, the VT code in [5] is the best code to correct a single deletion or insertion error and the modified VT code in [6] is the best code to correct a single deletion, insertion, or substitution error. To correct more than a single error, we briefly recap the binary code correcting at most two consecutive deletions from [7]. Moreover, we briefly present the deletion correction capability of the binary code in [7] when single deletion or two consecutive deletions occur.

**Definition** **1.**  *For 0≤d≤2n−1, the binary code C(n,2) in [7] with length n is given as*

(3) $$\begin{array}{c} C\left(n,2\right)=\{g\in F_{2}^{n}:Rsyn\left(0g\right)\equiv d\;\mathrm{mod}\;2n\}. \end{array}$$

The correction capability of the code in Definition 1 was also proved in [7]. From the length of the received sequences y, we can know that one or two consecutive bits are removed from the codeword g. If one deletion at the *j*-th bit or two consecutive deletions at the *j*-th and (j+1)-th bits occur, then y can be $g_{j}^{n-1}$ or $g_{j}^{n-2}$.

To determine the position of the deleted bit, we first calculate the difference of the run-syndrome as Δ = d−Rsyn(0y) mod 2n. If one deletion error occurs, Δ = $d-Rsyn(0g_{j}^{n-1})\;\mathrm{mod}\;2n$ and if two consecutive deletions occur, Δ = $d-Rsyn(0g_{j}^{n-2})\;\mathrm{mod}\;2n$. These values are used to identify the value and position *j* of the deleted bit if there is one deletion in the codeword g or the values and positions *j* and j+1 of the two deleted bits in the case that two consecutive deletions occur in the codeword g.

However, in the quaternary case, there exists the code to correct a single deletion or insertion error. The overview of *q*-ary insertion and deletion-correcting codes with length *n* is briefly presented in Definitions 2 and 3. The VT code family, known as the set of the most basic codes for correcting a single deletion or insertion, is defined as follows [15]:

**Definition** **2.**  *For 0≤a<n and 0≤e<q, the q-ary VT code with length n, $VT_{a,e}\left(n,q\right)$ is defined as*

(4) $$\begin{array}{cc} VT_{a,e}\left(n,q\right)\overset{\Delta }{=}\{c\in F_{q}^{n}: & \sum_{i=0}^{n}\left(i-1\right)\alpha_{i}\equiv a\;\mathrm{mod}\;n \end{array}$$

(5) $$\begin{array}{c} \sum_{i=0}^{n}c_{i}\equiv e\;\mathrm{mod}\;q\}, \end{array}$$

where $\alpha_{1}=1$ and $\alpha_{i}=\left\{\begin{array}{cc} 1, & if\;c_{i}\geq c_{i-1}\\ 0, & if\;c_{i}<c_{i-1} \end{array}\right.$ for 1<i≤n.

From Definition 2, since the binary sequence α = $\left(\alpha_{1},\alpha_{2},\ldots ,\alpha_{n}\right)$ is strongly related to the *q*-ary sequence c, a deletion of the *j*-th symbol in the codeword c also leads to a deletion of the *j*-th bit in the binary sequence α. Hence, from the help of the binary sequence α, the *q*-ary sequence is finally corrected.

Similarly, the authors of [16] proposed a single deletion-correcting code that defined the *q*-ary SVT code.

**Definition** **3.**  *For 0≤a≤P, 0≤e<q, and f∈{0,1}, the q-ary SVT code, $SVT_{a,e,f}\left(n,P,q\right)$, with length n is defined as*

(6) $$\begin{array}{cc} \;SVT_{a,e,f}\left(n,P,q\right)\overset{\Delta }{=}\;\{c\in F_{q}^{n}: & \sum_{i=1}^{n}i\alpha_{i}\equiv a\;\mathrm{mod}\;\left(P+1\right)\; \end{array}$$

(7) $$\begin{array}{c} \sum_{i=1}^{n}c_{i}\equiv e\;\mathrm{mod}\;q \end{array}$$

(8) $$\begin{array}{c} \sum_{i=1}^{n}\alpha_{i}\equiv f\;\mathrm{mod}\;2\}, \end{array}$$

where $\alpha_{1}=1$ and $\alpha_{i}=\left\{\begin{array}{cc} 1, & if\;c_{i}\geq c_{i-1}\\ 0, & if\;c_{i}<c_{i-1} \end{array}\right.$ for 1<i≤n.

Compared to the construction of the *q*-ary VT code, since mod (P+1) is used in the constraint (6) in Definition 3 instead of mod *n*, the redundancy of the *q*-ary SVT code is reduced from $\lceil log_{q}\left(n+1)\right\rceil$ to $\lceil log_{q}\left(2P+2\right)+1\rceil$. The constraint (8) is added to imply that the binary sequence α belongs to the binary SVT code. Hence, similar to the correcting method in Definition 2, the *q*-ary SVT code in Definition 3 can correct one deletion in any position.

However, the *q*-ary VT code in Definition 2 and the *q*-ary SVT code in Definition 3 correct only a single deletion or insertion error, but cannot correct consecutive deletions or insertions in the sequence. To solve this drawback, we can convert the idea in [8,9] about a construction for the binary codes correcting a burst of deletion or insertion errors with a size of exactly *b* for b≥2, into the *q*-ary case. The *q*-ary codeword c with length *n* is treated as a codeword array $A_{b}\left(c\right)$ with size $b\times \frac{n}{b}$ and the codeword is arranged column-by-column. Then, to reduce redundancy than in [8], the first row and each of the other (b−1) rows in the codeword array are encoded by a *q*-ary VT code and *q*-ary SVT code, respectively. From this construction, one deletion or insertion error in each row can be corrected by the *q*-ary VT code or *q*-ary SVT code, such that a burst of *b* consecutive deletion or insertion errors can be corrected.

For example, for correcting a burst of deletions of size two, the *q*-ary codeword c with length *n* is presented as a $2\times \frac{n}{2}$ array $A_{2}\left(c\right)$, which is given by

(9) $$\begin{array}{c} A_{2}\left(c\right)=\left[\begin{array}{cccc} c_{1} & c_{3} & \cdots & c_{n-1}\\ c_{2} & c_{4} & \cdots & c_{n} \end{array}\right]. \end{array}$$

Since each row of $A_{2}\left(c\right)$ is protected by the *q*-ary VT code or SVT code with length $\frac{n}{2}$, the code from $A_{2}\left(c\right)$ can correct exactly two consecutive deletions.

To sum up the previous statements, to correct one or exactly two consecutive deletion or insertion errors, we can use the *q*-ary VT and SVT codes. However, quaternary code to correct at most two consecutive deletion or insertion errors has not been developed. In the following section, we propose a new code design of quaternary codes suitable for the DNA storage and these codes can correct at most two consecutive deletion or insertion errors.

## 3. Proposed Code Design

This section provides a new design for a quaternary code to correct at most two consecutive deletions or insertions symbols. The construction of the proposed code is given in Section 3.1. Section 3.2 and Section 3.3 prove the correction capabilities of the presented code if one deletion occurs or two consecutive deletion errors occur, respectively. The decoding of insertion errors is presented in Section 3.4. The evaluation of a lower bound and an upper bound on the cardinality of the proposed code is derived in Section 3.5.

### 3.1. Code Construction

Exploring a new design for the quaternary code to correct one or two consecutive symbols, we explain the proposed code design as the following definition. In the proposed code design, the binary sequence which has the same length and related to the quaternary sequence is used to construct the constraints for the proposed code.

**Definition** **4.**  *For 0≤a≤n, 0≤d≤2n−1, and 0≤e<4, a quaternary code C(n,4) has a codeword c = $\left(c_{1},c_{2},\ldots ,c_{n}\right)$. First, we can consider a mapping from the quaternary codeword c to a binary sequence x = $\left(x_{1},x_{2},\ldots ,x_{n}\right)$ for 1≤i≤n as,*

(10) $$\begin{array}{c} x_{i}=\left\{\begin{array}{cc} 0, & if\;c_{i}=0\;or\;c_{i}=1\\ 1, & if\;c_{i}=2\;or\;c_{i}=3 \end{array}\right.. \end{array}$$

*Then, the quaternary code C(n,4) which satisfies the following three conditions can correct at most two consecutive deletion or insertion errors.*

(11) $$\begin{array}{cc} C\left(n,4\right)=\{c\in F_{4}^{n}: & Rsyn(0x)\equiv d\;\mathrm{mod}\;2n \end{array}$$

(12) $$\begin{array}{cc} \; & \sum_{i=1}^{n}ic_{i}\equiv a\;\mathrm{mod}\;\left(8n+1\right) \end{array}$$

(13) $$\begin{array}{cc} \; & \sum_{i=1}^{n}c_{i}\equiv e\;\mathrm{mod}\;4\}. \end{array}$$

The basic idea of the mapping (10) is that the quaternary codeword c corresponds to the binary sequence x with the same length *n*. Therefore, a deletion in the *j*-th position of the codeword c also leads to a deletion in the *j*-th position of the binary sequence x. For example, if the received sequence is y = $c_{j}^{n-1}\in F_{4}^{n-1}$, after using the mapping (10), we can obtain the binary sequence $x_{j}^{n-1}\in F_{2}^{n-1}$, which has one deletion error in the *j*-th position.

In Definition 4, the condition (11) is the same as the condition (3) in Definition 1 for C(n,2), which means that the sequence x is protected by a binary codeword of C(n,2). Therefore, decoding of the binary sequence x can be used for finding the positions of the deleted symbols and guessing the values of deleted symbols of codeword c.

The two constraints (12) and (13) in Definition 4, which are not in Definition 1, are used to obtain the correcting property in the quaternary regime. Since from constraint (11), the possible positions of the deletion errors can be obtained; however, in the case there is more than one value which satisfies the constraint (11), the constraints (12), (13) are used to remove invalid values of the possible positions. The constraint (13) is added to determine exactly the value of the deleted symbol and sum value of two consecutive deleted symbols. Then, finally the position and the value of symbols satisfy 3 constraints (11), (12) and (13) will be unique and the resulting quaternary sequence will be corrected. For example, n=10,d=0,a=0 and e=0, the binary sequence is corrected as $x=1\underline{10}0000111$, the underlined bits are the bits which are inserted to correct x. From the mapping (10), the possible quaternary sequence can be $c=0\underline{30}0011322$, $c=0\underline{20}0011322$, $c=0\underline{31}0011322$, or $c=0\underline{21}0011322$. If there are no constraints (12), (13), the decoder cannot output the corrected quaternary sequence. Therefore, the constraints (12), (13) exclude the invalid quaternary sequences as described in Table 1, then the output is the unique sequence $c=0\underline{30}0011322$.

### 3.2. Decoding Procedure for One Deletion Error

It is assumed that a transmitter and receiver share the parameters n,d,a,e of the code C(n,4) in Definition 4. Then, we first consider a case that one deletion error occurs in the codeword c. For 1≤i≤n, if the *j*-th symbol in c is removed, we obtain a received sequence $y=c_{j}^{n-1}\in F_{4}^{n-1}$, with length n−1.

If the symbol at the *j*-th position is deleted, the constraint (13) can be rewritten as $\sum_{i=1}^{j-1}c_{i}+c_{j}+\sum_{i=j+1}^{n}c_{i}\equiv e\;\mathrm{mod}\;4$. From the received sequence $y=c_{j}^{n-1}\in F_{4}^{n-1}$, the constraint is given as $\sum_{i=1}^{n-1}y_{i}=\sum_{i=1}^{j-1}y_{i}+\sum_{i=j}^{n-1}y_{i}=\sum_{i=1}^{j-1}c_{i}+\sum_{i=j+1}^{n}c_{i}$. Thus, the value of the deleted symbol value $c_{j}$ is calculated as $c_{j}=e-\sum_{i=1,i\neq j}^{n}c_{i}\;\mathrm{mod}\;4=e-\sum_{i=j}^{n-1}y_{i}\;\mathrm{mod}\;4$.

Next, we need to find the deletion position *j*. From the mapping (10) for the received sequence y to acquire the binary sequence $x_{j}^{n-1}$ with length n−1, $0x_{j}^{n-1}$ is obtained as $0x_{j}^{n-1}$ = $\left(0,x_{1},x_{2},\ldots ,x_{j-2},x_{j-1},x_{j+1},x_{j+2},\ldots ,x_{n}\right)$. Then, the run-length vector $r^{{}^{\prime }}$ is determined from $0x_{j}^{n-1}$ and $Rsyn(0x_{j}^{n-1})$ = $\sum_{i=0}^{∥r^{{}^{\prime }}∥-1}ir_{i}^{{}^{\prime }}\;\mathrm{mod}\;2n$ in the constraint (11). As mentioned in Definition 1, when one deletion error occurs, the run-syndrome decreases by Δ = $d-Rsyn(0x_{j}^{n-1})\;\mathrm{mod}\;2n$.

To provide a proof for the correction capabilities of the proposed quaternary code in Definition 4, we develop Algorithm 1 as a correcting method in the case of one deletion symbol.
**Algorithm 1** Correct one deletion symbol.**Input:** 
$n,d,a,e,y=c_{j}^{n-1}\in F_{4}^{n-1}$.**Output:** 
$c=\left(c_{1},c_{2},\ldots ,c_{n}\right)\in C\left(n,4\right)$. 1:$c_{j}=e-\sum_{i=j}^{n-1}y_{i}\;\mathrm{mod}\;4$. 2:Get the binary sequence $0x_{j}^{n-1}$ and the run-length vector $r^{{}^{\prime }}$ of $0x_{j}^{n-1}$. 3:Get the total number of elements of $r^{{}^{\prime }}$ as $∥r^{{}^{\prime }}∥$. 4:$\Delta =d-Rsyn(0x_{j}^{n-1})\;\mathrm{mod}\;2n$. 5:Set j=1. 6:**while** j≤n **do** 7:   **if** $\Delta <∥r^{{}^{\prime }}∥$ **then** 8:     **if** $k_{0x_{j}^{n-1}}\left(j-1\right)=\Delta$ **then** 9:        c=**del_correct1**$\left(n,a,y,j,c_{j}\right)$ 10:     **else** 11:        j←j+1 12:     **end if** 13:   **else** 14:     **if** $k_{0x_{j}^{n-1}}\left(j-1\right)+2\left(n-j\right)-1=\Delta$ **then** 15:        c=**del_correct1** $\left(n,a,y,j,c_{j}\right)$ 16:     **else** 17:        j←j+1 18:     **end if** 19:   **end if** 20:**end while**

Function 1 provides function **del_correct1** for Algorithm 1 to determine the deletion position, and then the output of Function 1 is the corrected quaternary sequence. In addition, in Function 1, *Syn_new* stands for the syndrome of the quaternary sequence after inserting the lost symbol $c_{j}$ in the *j*-th position of $c_{j}^{n-1}$.
**Function 1:** ***c*** = **del_correct1** (*n*, *a*, ***y***, *j*, *c_j_*)** Input:** 
*n*, *a*, ***y***, *j*, *c_j_*.**Output:** 
$c=\left(c_{1},c_{2},\ldots ,c_{n}\right)\in C\left(n,4\right)$.1:*Syn_new* = $\sum_{i=1}^{j-1}ic_{j,i}^{n-1}+jc_{i}+\sum_{i=j+1}^{n-1}ic_{j,i}^{n-1}$ mod (8*n* + 1)2:**if***Syn_new* = *a* **then**3:  ***c*** = *c_1_*,*c_2_*,…,*c_j−1_*,*c_j_*,*c_j + 1_*,…,*c_n_*4:**else**5:        j←j+16:**end if**

*Example 1*: Let n,d,a, and *e* be 10, 0, 0, and 0, respectively. Assume that one deletion occurs at the sixth position of the codeword c = $\left(0,3,0,0,0,1,1,3,2,2\right)\in F_{4}^{10}$. The received sequence y is y = $c_{j}^{9}$ = (0,3,0,0,0,1,3,2,2). As mentioned in Algorithm 1, the value of the lost symbol is $c_{j}$ = $e-\sum_{i=1}^{n-1}y_{i}\;\mathrm{mod}\;4=1$. From the mapping (10), we obtain the binary sequence $x_{j}^{9}$ = 010000111. Then, the run-length vector of $0x_{j}^{9}$ is $r^{{}^{\prime }}=\left(2,1,4,3\right)$ so $∥r^{{}^{\prime }}∥$ = 4 and the run-syndrome of $0x_{j}^{9}$ is $Rsyn(0x_{j}^{9})$ = $\sum_{i=0}^{5-1}ir_{i}^{{}^{\prime }}\;\mathrm{mod}\;20$ = 18. The change of the run-syndrome is computed as Δ = $0-Rsyn(0x_{j}^{9})\;\mathrm{mod}\;20=2$.

For 1≤j≤n, since $\Delta <∥r^{{}^{\prime }}∥$, following Algorithm 1, when *j* = 6, then Δ = $k_{0x_{j}^{9}}\left(j-1\right)$ = 2. If inserting the lost symbol with $c_{j}$ = 1 in the sixth position of the received sequence as $\left(0,3,0,0,0,\underline{1},1,3,2,2\right)$, the syndrome of this quaternary sequence Syn_new = $\sum_{i=1}^{6-1}ic_{6,i}^{9}+6.1+\sum_{i=6+1}^{10}ic_{6,i}^{9}\;\mathrm{mod}\;81$ = 0 (equals to *a*). Thus, the deletion error of the quaternary sequence is recovered correctly.

### 3.3. Decoding Procedure for Two Deletion Errors

Suppose that the received sequence y = $c_{j}^{n-1}\in F_{4}^{n-2}$ with length n−2, where two consecutive symbols in the *j*-th and (j+1)-th positions of codeword c∈C(n,4) are deleted.

The constraint (13) in Definition 4 can be rewritten as $\sum_{i=1}^{j-1}c_{i}+c_{j}+c_{j+1}+\sum_{i=j+2}^{n}c_{i}\equiv e\;\mathrm{mod}\;4$, and it is easy to obtain as $c_{j}+c_{j+1}$ = $e-\sum_{i=1,i\neq j,i\neq j+1}^{n}c_{i}\;\mathrm{mod}\;4$, corresponding to $c_{j}+c_{j+1}$ = $e-\sum_{i=1}^{n-2}c_{j,i}^{n-2}\;\mathrm{mod}\;4$. Since $\sum_{i=1}^{n-2}y_{i}$ = $\sum_{i=1}^{n-2}c_{j,i}^{n-2}$, we can rewrite $c_{j}+c_{j+1}$ as $c_{j}+c_{j+1}$ = $e-\sum_{i=1}^{n-2}y_{i}\;\mathrm{mod}\;4$.

From the mapping (10) for the received sequence y, the binary sequence with length n−1 is obtained as $0x_{j}^{n-2}$ = $\left(0,x_{1},x_{2},\ldots ,x_{j-2},x_{j-1},x_{j+2},x_{j+3},\ldots ,x_{n}\right)$. Then, the run-length vector r′′ of $0x_{j}^{n-2}$ also can determine the run-syndrome of $0x_{j}^{n-2}$ as $Rsyn(0x_{j}^{n-2})$ = $\sum_{i=0}^{∥r\prime \prime ∥-1}ir_{i}\prime \prime \;\mathrm{mod}\;2n$. Thus, similar to the approach mentioned in Definition 1, the difference of the run-syndrome is computed as Δ = $d-Rsyn(0x_{j}^{n-2})\;\mathrm{mod}\;2n$.

To recover two deletion errors, we first recover the binary sequence x with length *n* from the binary sequence $x_{j}^{n-2}$. The authors of [7] suggested the eight possible instances when two consecutive bits are deleted, as summarized in Table 2. However, in this work, we consider more instances which are 16 in total, and the remaining eight instances are listed in Table 3. Please note that in Algorithms 2 and A2, a notation $x_{j}={\bar{x}}_{j-1}$ is used to imply that the reverse value of the (j−1)-th position is assigned to the bit at the *j*-th position. Thus, two notations $x_{j}={\bar{x}}_{j-1}$ and $x_{j}\neq x_{j-1}$ have the same meaning, and this means that two neighbor (j−1)-th and *j*-th bits have different values. For example, if $x_{j-1}=1$, then $x_{j}={\bar{x}}_{j-1}=0$ or $x_{j}\neq x_{j-1}=0$.

In an analysis approach similar to [7], for 1≤j≤n−1, there are four possible deleted bit pairs $\left(x_{j},x_{j+1}\right)$ = (0,0),(0,1),(1,0), and (1,1). Then, if j+2 ≤n, we combine four possible cases of $\left(x_{j},x_{j+1}\right)$ with neighboring bits $\left(x_{j-1},x_{j+2}\right)$ = (0,0),(0,1),(1,0), and (1,1), and we need to consider 16 instances of $\left(x_{j-1},x_{j},x_{j+1},x_{j+2}\right)$.

From the above analysis, we develop Algorithm 2 for the proposed code to correct two consecutive deletion errors. In addition, in Algorithm 2, though it was not mentioned, the bit $x_{j+2}$ is mathematically analyzed as an accompanied pair with $x_{j-1}$, as described above, to obtain the conditions, such as lines 9, 16, 27, 34 to determine the deleted positions.
**Algorithm 2** Correct two consecutive deletion symbols**Input:** 
$n,d,a,e,y=c_{j}^{n-2}\in F_{4}^{n-2}$.**Output:** 
$c=\left(c_{1},c_{2},\ldots ,c_{n}\right)\in C\left(n,4\right)$. 1:$c_{j}+c_{j+1}=e-\sum_{i=j}^{n-2}y_{i}\;\mathrm{mod}\;4$. 2:Get the binary sequence $0x_{j}^{n-2}$ and the run-length vector r′′ of $0x_{j}^{n-2}$. 3:Get the total number of elements of r′′ as ∥r′′∥. 4:Δ = $d-Rsyn(0x_{j}^{n-2})\;\mathrm{mod}\;2n$. 5:Set j=1. 6:**if** Δ≥2∥r′′∥ **then** 7:   **while** j≤n−1 **do** 8:     **if** mod(Δ,2)=1 **then** 9:        **if** $2k_{0x_{j}^{n-2}}\left(j-1\right)+2\left(n-j\right)+1=\Delta$ **then** 10:          $x_{j}={\bar{x}}_{j-1}$; $x_{j+1}=x_{j-1}$ 11:          c = **del_correct2**$(n,a,y,j,x_{j},x_{j+1},c_{j}$+$c_{j+1})$ 12:        **else** 13:          j←j+1 14:        **end if** 15:     **else** 16:        **if** $2k_{0x_{j}^{n-2}}\left(j-1\right)+2\left(n-j\right)=\Delta$ **then** 17:          $x_{j}={\bar{x}}_{j-1}$; $x_{j+1}={\bar{x}}_{j-1}$ 18:          c = **del_correct2**$(n,a,y,j,x_{j},x_{j+1},c_{j}$+$c_{j+1})$ 19:        **else** 20:          j←j+1 21:        **end if** 22:     **end if** 23:   **end while** 24:**else** 25:   **while** j≤n−1 **do** 26:     **if** mod(Δ,2)=1 **then** 27:        **if** $2k_{0x_{j}^{n-2}}\left(j-1\right)+1=\Delta$ **then** 28:          $x_{j}=x_{j-1}$; $x_{j+1}={\bar{x}}_{j-1}$ 29:          c = **del_correct2**$(n,a,y,j,x_{j},x_{j+1},c_{j}$+$c_{j+1})$ 30:        **else** 31:          j←j+1 32:        **end if** 33:     **else** 34:        **if** $2k_{0x_{j}^{n-2}}\left(j-1\right)=\Delta$ **then** 35:          $x_{j}=x_{j-1}$; $x_{j+1}=x_{j-1}$ 36:          c = **del_correct2**$(n,a,y,j,x_{j},x_{j+1},c_{j}$+$c_{j+1})$ 37:        **else** 38:          j←j+1 39:        **end if** 40:     **end if** 41:   **end while** 42:**end if**

To clarify the explanation of the function **del_correct2** for Algorithm 3 in Section 3.3, we provide the detail in Function 2. In Function 2, *Syn_new* implies the syndrome of the quaternary sequence after inserting the lost symbols $c_{j}$ and $c_{j+1}$ in the *j*-th and (j+1)-th position of $c_{j}^{n-2}$. If the value of *Syn_new* equals to the parameter of syndrome *a*, we infer that the quaternary sequence is retrieved successful.
**Function 2:** ***c*** = **del_correct2** (*n*, *a*, ***y***, *j*, *c_j_* + *c*_*j*+1_)** Input:** 
*n*, *a*, ***y***, *j*, *x_j_*, *x*_*j*+1_, *c_j_* + *c*_*j*+1_.**Output:** 
$c=\left(c_{1},c_{2},\ldots ,c_{n}\right)\in C\left(n,4\right)$.1:Using mapping (10) to obtain *c_j_*, then *c*_*j*+1_ = *c_j_* + *c*_*j*+1_ − *c*_*j*_2:*Syn_new* = $\sum_{i=1}^{j-1}ic_{j,i}^{n-2}+jc_{i}+{\left(j+1\right)}_{c_{j+1}}+\sum_{i=j+2}^{n-2}ic_{j,i}^{n-2}$ mod (8*n* + 1)3:**if***Syn_new* = *a* **then**4:  *c* = *c_1_*,*c_2_*,…,*c_j−1_*,*c_j_*,*c_j + 1_*,…,*c_n_*5:**else**6:        j←j+17:**end if**

*Example 2*: Let n,d,a, and *e* be 10, 0, 0, and 0, respectively. It is assumed that two consecutive deletions occur at the seventh and eighth position of the codeword c = $\left(0,3,0,0,0,1,1,3,2,2\right)\in F_{4}^{10}$ and the received the quaternary sequence is y = $c_{j}^{8}$ = (0,3,0,0,0,1,2,2). As mentioned in Algorithm 3, the sum of the values of the two deleted symbols is $c_{j}+c_{j+1}$ = $e-\sum_{i=1}^{n-2}y_{i}\;\mathrm{mod}\;4$ = $e-\sum_{i=1}^{n-2}c_{j,i}^{8}\;\mathrm{mod}\;4$ = 0.

From the mapping (10) for $c_{j}^{8}$, the binary sequence $0x_{j}^{8}$ and r′′ are $0x_{j}^{8}$ = 001000011 and r′′ = (2,1,4,2), respectively. Then, ∥r′′∥ = 4 and the run-syndrome $Rsyn(0x_{j}^{8})$ = 15. The difference of run-syndrome is calculated as Δ = $0-Rsyn(0x_{j}^{8})\;\mathrm{mod}\;20$ = 5.

From Algorithm 2, since Δ<2∥r′′∥ and Δ mod 2=1, for 1≤j≤n−1, the value j=7 satisfies the equation Δ = $2k_{0x_{j}^{8}}\left(j-1\right)+1$ = $2k_{0x_{j}^{8}}\left(6\right)+1$ = 5. Thus, as mentioned in line 28 of Algorithm 2, we obtain $x_{7}=0$ and $x_{8}=1$, and the corrected binary sequence is 0100000111.

Applying mapping (10) to the binary sequence $010000\underline{01}11$ and $c_{j}+c_{j+1}=0$, the two deleted symbols $\left(c_{7},c_{8}\right)$ are determined as (1,3). The syndrome *Syn_new* of the quaternary sequence when inserting $\left(c_{7},c_{8}\right)$= (1,3) into $c_{j}^{8}$ is 0, which equals the syndrome a of codeword c. Thus, finally the recovered quaternary sequence is $\left(0,3,0,0,0,1,\underline{1},\underline{3},2,2\right)$.

Algorithms 1 and 2 are proved using an exhaustive search strategy to show that the proposed code can correct at most two consecutive deletion symbols. However, as mentioned in [9], deletion-correcting codes are not always successful in identifying the exact location of the deleted symbols. For example, if an all-zero codeword is sent and one deletion error occurs, to find value of the deleted symbol is easy but it is impossible to find the exact position of the deleted symbol. Even though the exact position cannot be detected, the codeword can be successfully recovered by inserting a zero symbol in any position. This means that when the exact index of the deleted error is not detected but the run index which the deleted error belongs to is determined, the codeword can be successfully recovered by inserting one symbol in any position in the run.

If a codeword with a large run was sent and one deletion occurs in the large run, the proposed algorithm can always determine the value and the run index of the deleted symbol but rarely find the exact position of the deleted symbol in the run. In this case, we prioritize the proposed algorithm to output the first index in the detected run. Therefore, when a deletion error occurs in a large run and it is not possible to find the exact position in a codeword, the codeword of the proposed code will be successfully decoded by inserting the deleted symbol in the first index of the run.

### 3.4. Decoding Procedure for Insertion Errors

Since there is a similarity to the case of deletion errors, in this subsection, the correction capability of this proposed code for insertion errors is briefly presented. The received quaternary sequence y has a length that is one or two symbols larger than *n*, if one or two consecutive insertion errors occur. Table 4 summarizes the different computations of decoding between insertion and deletion errors.

#### 3.4.1. Correcting one Insertion Error

It is assumed that the received sequence with length n+1 is y = $c_{j}^{n+1}$ = $\left(c_{1},c_{2},\ldots ,c_{j-1},h_{1},c_{j},c_{j+1},\ldots ,c_{n}\right)\in F_{4}^{n+1}$, this means that one symbol $h_{1}$ is inserted at the *j*-th position of the codeword c∈C(n,4). The process to correct the received sequence y can be briefly presented by the following steps.

The first step is calculating the value of the inserted symbol $h_{1}$ in y. The received sequence y has a sum of total symbols computed as $\sum_{i=1}^{n+1}y_{i}$ = $\sum_{i=1}^{n+1}c_{j,i}^{n+1}$ = $\sum_{i=1}^{j-1}c_{i}+h_{1}+\sum_{i=j}^{n}c_{i}$, and then $\sum_{i=1}^{n+1}c_{j,i}^{n+1}\;\mathrm{mod}\;4$ = $\sum_{i=1}^{n}c_{i}+h_{1}\;\mathrm{mod}\;4$ = $e+h_{1}\;\mathrm{mod}\;4$. The value of the inserted symbol $h_{1}$ is calculated by $h_{1}$ = $\sum_{i=1}^{n+1}c_{j,i}^{n+1}-e\;\mathrm{mod}\;4$.

The second step is determining the insertion position *j*. From mapping (10), we obtain the binary sequence $0x_{j}^{n+1}$. From the binary sequence $0x_{j}^{n+1}$, we obtain the run-length vector of $0x_{j}^{n+1}$ and then calculate the difference of the run-syndrome by Δ = $Rsyn(0x_{j}^{n+1})-d\;\mathrm{mod}\;2n$. To determine the position *j* of the inserted symbol $h_{1}$, in Appendix A, we provide Algorithm A1 and Function 3 for this step and the output is the corrected quaternary sequence.

#### 3.4.2. Correcting Two Consecutive Insertion Errors

If two consecutive insertion errors occur at the *j*-th and (j+1)-th positions of the codeword c∈C(n,4), the received sequence is y = $c_{j}^{n+2}$= $(c_{1},c_{2},\ldots ,c_{j-1},h_{1},h_{2},c_{j},c_{j+1},\ldots ,$$c_{n})\in F_{4}^{n+2}$ with length n+2. From the received sequence $c_{j}^{n+2}$ and a similar analysis as the one insertion case, the sum of the two inserted symbols is obtained as $h_{1}+h_{2}$ = $\sum_{i=1}^{n+2}c_{j,i}^{n+2}-e\;\mathrm{mod}\;4$ = $\sum_{i=1}^{n+2}y_{i}-e\;\mathrm{mod}\;4$.

From the mapping (10) in Definition 4, since two consecutive symbols $h_{1},h_{2}$ are inserted in $c_{j}^{n+2}$ corresponding two consecutive bits are also inserted in the binary sequence $x_{j}^{n+2}$, we can obtain the binary sequence $0x_{j}^{n+2}$. Thus, the run-syndrome of $0x_{j}^{n+2}$ is calculated by Δ = $Rsyn(0x_{j}^{n+2})-d\;\mathrm{mod}\;2n$. Algorithm A2 and Function 4 in Appendix B are provided to determine exact values of $h_{1},h_{2}$ and the positions *j* and j+1 of the two consecutive insertion errors. Finally, $h_{1}$ and $h_{2}$ are removed from the sequence $c_{j}^{n+2}$ to retrieve the codeword c.

### 3.5. Cardinality of the Proposed Code

Since our main contribution is the correction code capability of the proposed code, then the lower bounds and upper bound of this code design is evaluated based on the previous methods in [7,15].

#### 3.5.1. Lower Bound of the Code Cardinality

In [15] of Section IV, the lower bound of the code cardinality was determined by the potential values of the syndrome and checksum in the code construction. Hence, with the similar approach, by applying d∈[0,2n−1],a∈[0,8n] and e∈[0,3], we can obtain the lower bound for the cardinality m(n,4) of the proposed code as

(14) $$\begin{array}{c} m\left(n,4\right)\geq \frac{4^{n}}{8n(8n+1)}. \end{array}$$

The redundancy of the proposed code can be at most as below

(15) $$\begin{array}{c} n-\log_{4}|C(n,4)|\leq n-\log_{4}\frac{4^{n}}{8n(8n+1)}\approx 2\log_{4}8n. \end{array}$$

#### 3.5.2. Upper Bound of the Code Cardinality

Define |M(n,4)| as the cardinality of the quaternary code of length *n*, with a maximum possible number of codewords, which can correct at most two consecutive deletion or insertion errors. Similar to the method in [7], the upper bound of the cardinality of |M(n,4)| is evaluated as

(16) $$\begin{array}{c} \left|M(n,4)|\leq \right|M_{1}\left(n,4\right)\left|+\right|M_{2}\left(n,4\right)|. \end{array}$$

where $|M_{1}\left(n,4\right)|$ is the number of codewords with length *n* such that the number of runs is larger than (r+1) (with *r* is an arbitrary number) and $|M_{2}\left(n,4\right)|$ is number of codewords with length *n* such that the number of runs is not larger than (r+1). The Equation (16) will be represented as

(17) |M(n,4)|≤4n−22(r+1)+1+2.4n−⌊n−22⌋∑j=0r⌊n−22⌋j.

Let we set *r*=$\left\lfloor \frac{\lfloor 3\left\lfloor \frac{n-2}{2}\right\rfloor -\sqrt{2\left\lfloor \frac{n-2}{2}\right\rfloor \ln\left\lfloor \frac{n-2}{2}\right\rfloor }}{4}\right\rfloor$, and let *n* tends to infinity, then $2\left(r+1\right)+1\approx \frac{3}{2}n$. Therefore, with $r\leq \frac{3\lfloor \frac{n-2}{2}\rfloor }{4}$, the upper bound of the cardinality of the proposed code can be written as

(18) $$\begin{array}{c} \left|M(n,4)\right|\leq \frac{2\;\cdot \;4^{n-2}}{3n}. \end{array}$$

## 4. Discussion

In this section, we explain the results of our proposed code design and then discuss about the applications of the proposed code.

We provide a new design of quaternary codes to correct at most two consecutive deletion or insertion errors. From Algorithms 1 and 2 and Appendixes Appendix A and Appendix B the correction capabilities of this design with deletion and insertion errors are proved. Obviously, with this proposed code, we can consider 0↔A, 1↔C, 2↔T, and 3↔G to directly construct or sequencing the DNA strands.

To deal with a burst of size of at most *b* (for any fixed b≥3) deletion or insertion errors, the intersection between the proposed code and the quaternary code can correct exactly b−2 consecutive deletion or insertion errors.

For example, to correct a burst error of a size of at most b=3, first, we create the code C(n,4) from Definition 4, which takes care of one or two consecutive deletion or insertion errors. Then, we create a *q*-ary VT code $VT_{a,e}\left(n,4\right)$ from Definition 2 with q=4 to correct a single error. Then, by intersecting C(n,4) and $VT_{a,e}\left(n,4\right)$ we can obtain the expected quaternary code with length *n*, to deal with a burst error of size at most three. With given b>3, we use the array code construction which is described in Section 2.2 to create a quaternary code that can correct exactly b−2 consecutive deletion or insertion errors. Through intersection of this code and our proposed code, a quaternary code that can correct at most b>3 consecutive deletion or insertion errors can be obtained.

## 5. Conclusions

In this paper, we propose a new design of a quaternary code to correct at most two consecutive deletion or insertion errors with redundancy at most $2\log_{4}8n$ symbols. We also develop decoding algorithms for correcting one and two consecutive deletion or insertion errors in any quaternary sequences. Even though the results in this work provide significant applications for DNA storage and correction of multiple quaternary errors, there are still several open problems, such as code constructions which can correct at most *b* non-consecutive deletion or insertion errors and codes that can correct at most *b* deletion or insertion and substitution errors, for arbitrary *b*. Moreover, the optimal design when concatenation of constrained code and our proposed code for DNA-based data storage also needs to be considered.

## Figures and Tables

**Table 1:** Correction capability of constraints (12), (13) when two consecutive deletions occur.

Possible Corrected Quaternary Sequences	Compared to *a* in Constraint (12)	Compared to *e* in Constraint (13)
0300011322	O	O
0200011322	X	X
0310011322	X	X
0210011322	X	O

**Table 2:** The eight possible instances in [7] for two consecutive deletion errors.

Conditions of $\left(x_{j-1},x_{j},x_{j+1},x_{j+2}\right)$ in [7]	$\left(x_{j-1},x_{j},x_{j+1},x_{j+2}\right)$ in [7]
$x_{j}=x_{j-1},x_{j+1}=x_{j-1},x_{j+2}\neq x_{j-1}\;\mathrm{if}\;j+2\leq n$	(1,1,1,0)
	(0,0,0,1)
$x_{j}=x_{j-1},x_{j+1}\neq x_{j-1},x_{j+2}\neq x_{j-1}\;\mathrm{if}\;j+2\leq n$	(1,1,0,0)
	(0,0,1,1)
$x_{j}\neq x_{j-1},x_{j+1}\neq x_{j-1},x_{j+2}=x_{j-1}\;\mathrm{if}\;j+2\leq n$	(1,0,0,1)
	(0,1,1,0)
$x_{j}\neq x_{j-1},x_{j+1}=x_{j-1},x_{j+2}=x_{j-1}\;\mathrm{if}\;j+2\leq n$	(0,1,0,0)
	(1,0,1,1)

**Table 3:** The eight possible instances are added in this work for two consecutive deletion errors.

Conditions of $\left(x_{j-1},x_{j},x_{j+1},x_{j+2}\right)$	$\left(x_{j-1},x_{j},x_{j+1},x_{j+2}\right)$
$x_{j}=x_{j-1},x_{j+1}=x_{j-1},x_{j+2}=x_{j-1}\;\mathrm{if}\;j+2\leq n$	(1,1,1,1)
	(0,0,0,0)
$x_{j}=x_{j-1},x_{j+1}\neq x_{j-1},x_{j+2}=x_{j-1}\;\mathrm{if}\;j+2\leq n$	(1,1,0,1)
	(0,0,1,0)
$x_{j}\neq x_{j-1},x_{j+1}\neq x_{j-1},x_{j+2}\neq x_{j-1}\;\mathrm{if}\;j+2\leq n$	(1,0,0,0)
	(0,1,1,1)
$x_{j}\neq x_{j-1},x_{j+1}=x_{j-1},x_{j+2}\neq x_{j-1}\;\mathrm{if}\;j+2\leq n$	(0,1,0,1)
	(1,0,1,0)

**Table 4:** The differences between insertion and deletion errors.

Content	One Insertion Error	Two Consecutive Insertion Errors	One Deletion Error	Two Consecutive Deletion Errors
Length of the received sequence	n+1	n+2	n−1	n−2
Sum of error symbol(s)	$\sum_{i=1}^{n+1}y_{i}-e\;\mathrm{mod}\;4$	$\sum_{i=1}^{n+2}y_{i}-e\;\mathrm{mod}\;4$	$e-\sum_{i=1}^{n-1}y_{i}\;\mathrm{mod}\;4$	$e-\sum_{i=1}^{n-2}y_{i}\;\mathrm{mod}\;4$
Difference of run-syndrome (mod 2*n*)	$Rsyn(0x_{j}^{n+1})-d$	$Rsyn(0x_{j}^{n+2})-d$	$d-Rsyn(0x_{j}^{n-1})$	$d-Rsyn(0x_{j}^{n-2})$

## Data Availability

Not applicable.

## Abbreviations

   The following abbreviations are used in this manuscript:

DNA	Deoxyribonucleic acid
VT	Varshamov–Tenengolts
SVT	shifted-Varshamov–Tenengolts
GC-content	Guanine-Cytosine content
MILP	Mixed Integer Linear Programming

## Appendix A. One Insertion Error Correction

As mentioned in Section 3.4, Algorithm A1 and the function **ins1_correct1** which is used in Algorithm A1 are given as follows.

**Algorithm A1** Correct one insertion symbol
Input: $n,a,y,0x_{j}^{n+1}$. Output: $c=\left(c_{1},c_{2},\ldots ,c_{n}\right)\in C\left(n,4\right)$. 1: Calculate Δ and $h_{1}$ as in Table 3. 2: Set j=1. 3: **while** j≤n+1 **do** 4: **if** $\Delta <∥r^{{}^{\prime }}∥$ **then** 5: **if** mod(Δ,2)=1 **then** 6: **if** $k_{0x_{j}^{n+1}}\left(j-1\right)+1=\Delta$ **then** 7: c=**ins1_correct1**$\left(n,a,y,j,h_{1}\right)$ 8: **else** 9: j←j+1 10: **end if** 11: **else** 12: **if** $k_{0x_{j}^{n+1}}\left(j-1\right)=\Delta$ **then** 13: c=**ins1_correct1**$\left(n,a,y,j,h_{1}\right)$ 14: **else** 15: j←j+1 16: **end if** 17: **end if** 18: **else** 19: **if** $k_{0x_{j}^{n+1}}\left(j-1\right)+2\left(n-j+1\right)+1=\Delta$ **then** 20: c=**ins1_correct1**$\left(n,a,y,j,h_{1}\right)$ 21: **else** 22: j←j+1 23: **end if** 24: **end if** 25: **end while**

Function 3: ***c*** = **ins1_correct1** (*n*, *a*, ***y***, *j*, *h*_1_) Input: *n*, *a*, ***y***, *j*, *h*_1_. Output: $c=\left(c_{1},c_{2},\ldots ,c_{n}\right)\in C\left(n,4\right)$. 1: **if**$c_{j}^{n+1}$ (*j*) = *h*_1_ **then** 2: *Syn_new* = $\sum_{i=1}^{j+1}ic_{j,i}^{n-1}+\sum_{i=j+1}^{n+1}(i-1)c_{j,i}^{n+1}$ mod (8*n* + 1) 3: **else** 4: j←j+1 5: **end if** 6: **if***Syn_new* = *a* **then** 7: ***c*** = *c_1_*,*c_2_*,…,*c_j−1_*,*c_j_*,*c_j + 1_*,…,*c_n_* 8: **else** 9: j←j+1 10: **end if**

Algorithm A1 finds the possible position *j* of the inserted symbol as steps 6, 13, 19 then uses Function 3 which presents function **ins1_correct1** to check this value of *j* to satisfy the constraint (12).

To determine the position of the inserted symbol, the value of *Syn_new* in line 2 of Function 3 indicates the syndrome of the received quaternary sequence $c_{j}^{n+1}$ when not considering the inserted symbol $c_{j}^{n+1}\left(j\right)$. Thus, in the second term of the right-hand side, the coefficient needs to be (i−1). This syndrome is compared to the constraint (12) to obtain the position of the inserted symbols and finally, remove the inserted symbol in the *j*-th position in $c_{j}^{n+1}$. Therefore, the quaternary sequence satisfies constraints (11), (12), and (13) can correct any one insertion error.

## Appendix B. Two Consecutive Insertion Errors Correction

To correct the quaternary sequence when two consecutive insertion errors occur, we provide the details of correction procedure in Algorithm A2 and Function 4.

Algorithm A2 is constructed based on the analysis which is mentioned in Section 3.5. From steps 6, 13, 24, 31, the possible positions *j* and j+1 of two consecutive insertion errors in the related binary sequence x can be obtained. However, since mapping (10) is used to map from quaternary symbols to binary bits, there can exist different cases of quaternary symbols which are mapped to the same binary bits, so we need to verify the exact quaternary values corresponding to the *j*-th and (j+1)-th positions.

Function 4: ***c*** = **ins2_correct2** (*j*, *n*, *a*, ***y***, *h*_1_ + *h*_2_) Input: *j*, *n*, *a*, ***y***, *h*_1_ + *h*_2_. Output: $c=\left(c_{1},c_{2},\ldots ,c_{n}\right)\in C\left(n,4\right)$. 1: **if**$c_{j}^{n+2}$ (*j*) = *h*_1_ **and** $c_{j}^{n+2}$ (*j* + 1) = *h*_1_ + *h*_2_ − *h*_1_ **then** 2: *Syn_new* = $\sum_{i=1}^{j-1}ic_{j,i}^{n+2}+\sum_{i=j+2}^{n+2}(i-2)c_{j,i}^{n+2}$ mod (8*n* + 1) 3: **else** 4: j←j+1 5: **end if** 6: **if***Syn_new* = *a* **then** 7: ***c*** = *c_1_*,*c_2_*,…,*c_j−1_*,*c_j_*,*c_j + 1_*,…,*c_n_* 8: **else** 9: j←j+1 10: **end if**

The function **ins2_correct2** in Algorithm A2 is provided as Function 4 to output the unique sequence which satisfies three constraints (11), (12), and (13). Steps 1,2 in Function 4 correspond to the comparison to the constraints (13) and (12), respectively. This comparison determines the exact value and position of the inserted symbols as mentioned in Section 3.1.

In the similar way to Function 3 in Appendix A, the function *Syn_new* calculates syndrome of $c_{j}^{n+2}$ when not considering the symbols in the *j*-th and (j+1)-th positions. This leads to the coefficient in the second term of function *Syn_new* is (i−2), meaning that the symbols which are after the (j+1)-th symbols are shifted to the left by 2 positions. The syndrome value *Syn_new* is compared to the value *a* of the constraint (12) to determine the exact values and positions of the inserted symbols of sequence. Obviously, the output sequence will satisfy both three constraints in Definition 4. Finally, two consecutive inserted symbols at the *j*-th and (j+1)-th positions of $c_{j}^{n+2}$ are removed. The output of Function 4 is the corrected quaternary sequence.

**Algorithm A2:** Correct two consecutive insertion symbols
Input: $n,a,y,0x_{j}^{n+2}$. Output: $c=\left(c_{1},c_{2},\ldots ,c_{n}\right)\in C\left(n,4\right)$. 1: Calculate Δ and $h_{1}+h_{2}$ as in Table 3. 2: Set j=1. 3: **if** Δ≥2∥r′′∥ **then** 4: **while** j≤n+1 **do** 5: **if** mod(Δ,2)=1 **then** 6: **if** $2k_{0x_{j}^{n+2}}\left(j-1\right)+2\left(n-j\right)+5=\Delta$ **then** 7: $x_{j}={\bar{x}}_{j-1}$; $x_{j+1}=x_{j-1}$ 8: c=**ins2_correct2**$\left(j,n,a,y,h_{1}+h_{2}\right)$ 9: **else** 10: j←j+1 11: **end if** 12: **else** 13: **if** $2k_{0x_{j}^{n+2}}\left(j-1\right)+2\left(n-j\right)+4=\Delta$ **then** 14: $x_{j}={\bar{x}}_{j-1}$; $x_{j+1}={\bar{x}}_{j-1}$ 15: c=**ins2_correct2**$\left(j,n,a,y,h_{1}+h_{2}\right)$ 16: **else** 17: j←j+1 18: **end if** 19: **end if** 20: **end while** 21: **else** 22: **while** j≤n+1 **do** 23: **if** mod(Δ,2)=1 **then** 24: **if** $2k_{0x_{j}^{n+2}}\left(j-1\right)+1=\Delta$ **then** 25: $x_{j}=x_{j-1}$; $x_{j+1}={\bar{x}}_{j-1}$ 26: c=**ins2_correct2**$\left(j,n,a,y,h_{1}+h_{2}\right)$ 27: **else** 28: j←j+1 29: **end if** 30: **else** 31: **if** $2k_{0x_{j}^{n+2}}\left(j-1\right)=\Delta$ **then** 32: $x_{j}=x_{j-1}$; $x_{j+1}=x_{j-1}$ 33: c=**ins2_correct2**$\left(j,n,a,y,h_{1}+h_{2}\right)$ 34: **else** 35: j←j+1 36: **end if** 37: **end if** 38: **end while** 39: **end if**
